# Evaluating the Effects of Acquisition and Search Parameters on Repository-Scale MS/MS Matching

**DOI:** 10.1021/jasms.6c00332

**Published:** 2026-10-23

**Authors:** Allison J. Keys, Emily C. Gentry

**Affiliations:** Department of Chemistry, 1757 Virginia Tech, Blacksburg, Virginia 24061, United States

**Keywords:** tandem mass spectrometry, metabolomics, spectral matching, benchmarking, meta-analysis

## Abstract

Tandem mass spectrometry (MS/MS) spectral matching is increasingly performed at the repository scale, where tools such as MASST enable a query spectrum to be compared against heterogeneous public data. Unlike analogous genomic-based searches, repository-scale MS/MS matching lacks integrated false discovery rate (FDR) reporting, leaving parameter selection entirely to user discretion. Here, we evaluate how acquisition factors and analysis filters jointly affect MS/MS matching using a curated library of 40 metabolites spanning ten chemical classes. In this study, we find that normalized collision energies from 30 to 50% balance sufficient fragmentation against loss of larger diagnostic ions, producing the most structurally informative spectra and yielding the highest proportion of repository matches. Using a fragmentation-based decoy library, we show that six minimum matching peaks achieve <1% FDR across the entire cosine threshold range tested (0.7–1.0), while fewer matching peaks require increasingly stringent cosine thresholds. These findings provide practical, benchmarking guidance for user-provided acquisition and search parameters in MASST searches.

## Graphical abstract

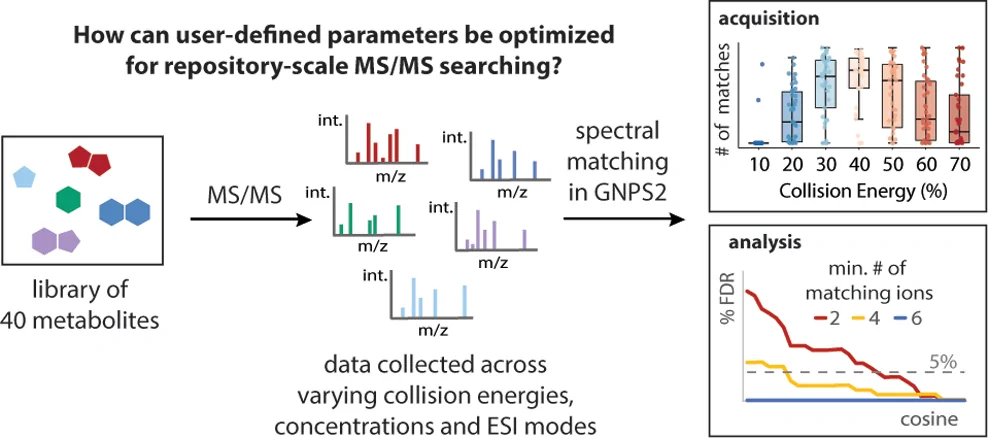

## Introduction

Tandem mass spectrometry (MS/MS) is influenced by a variety of user-selected factors, including instrument type, collision energy and ionization mode, producing many unique spectra for a single molecule (Figure 1a).^1–4^ Differences in mass analyzers affect ion transmission and fragmentation mechanisms, collision energy directly governs the extent of fragmentation and sample concentration determines ion abundance.^5–7^ Additional user-defined acquisition variables such as width of isolation window, solvent and spray voltage can also affect MS/MS spectra.^8–10^ Collectively, these experimental factors explain why significant spectral differences are observed for the same molecule under varying conditions.

**Figure 1:**
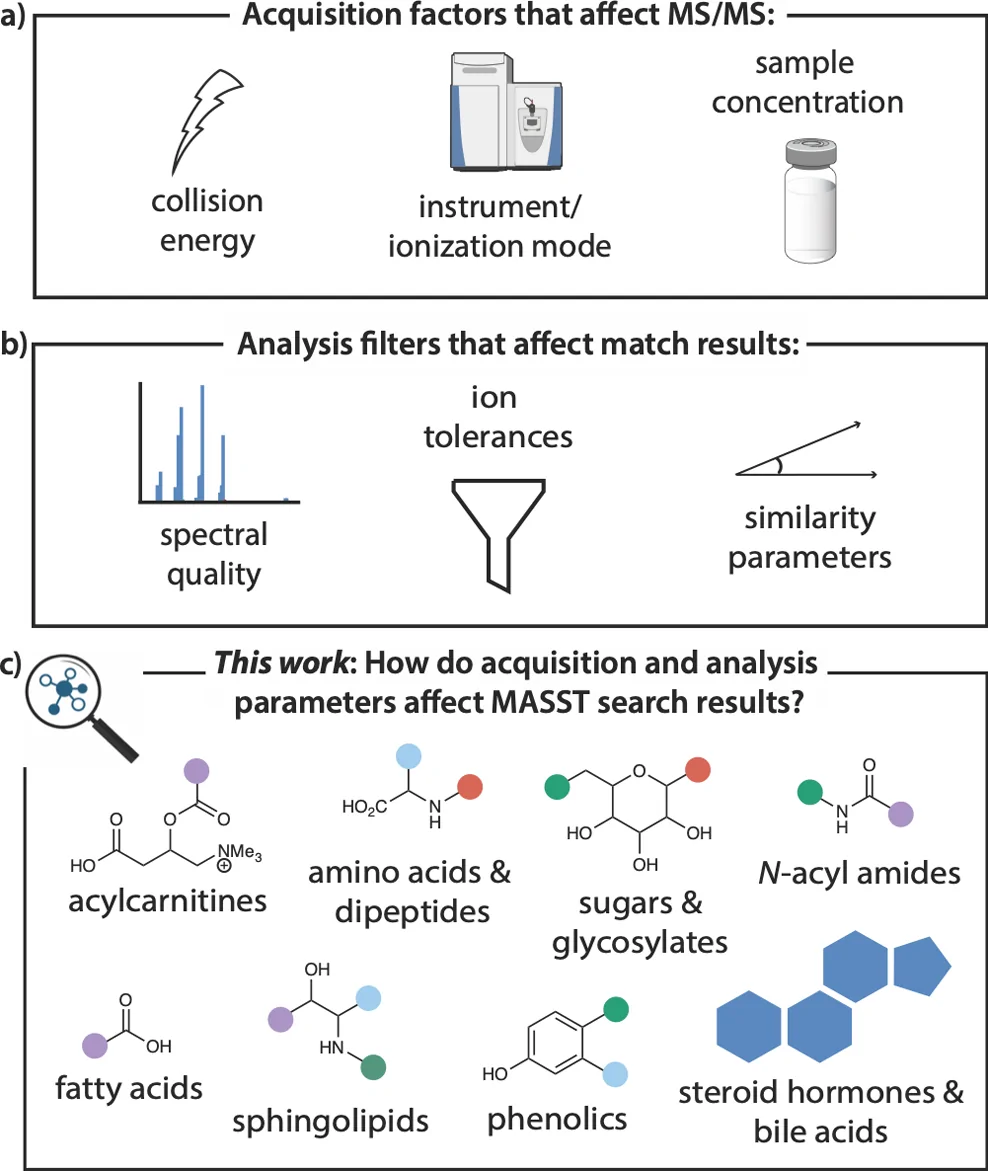
Examining LC–MS/MS parameters that affect spectral matching during (a) acquisition and (b) subsequent analysis. (c) This work explores the impact of acquisition and analysis parameters on repository-scale MS/MS searching using a diverse library of 40 common metabolites from animals, plants and microbes.

These variances have direct consequences for MS/MS spectral matching in untargeted metabolomics, as library searching relies on similarity of MS/MS spectra acquired across instrument platforms and collision energies.^11,12^ Moreover, repository-scale searching is increasing in popularity and depends on the comparison of spectra acquired from different laboratories across the world.^13^ In both of these workflows, users provide analysis thresholds such as cosine values and minimum number of matched fragment ions, which define the quality of a spectral match (Figure 1b). Since cosine similarity represents each MS/MS spectrum as a vector of fragment ion intensities, and collision energy directly influences these intensities, spectra of the same molecule acquired at different collision energies can yield low similarity scores. Matching thresholds must therefore strike a balance: criteria that are too strict miss true matches acquired under different conditions, while criteria that are too lenient allow for false positives. Previous approaches have estimated false discovery rates (FDR) for MS/MS matching of small molecules across data sets and in some cases, recommended matching thresholds accordingly.^14–16^ Cross-platform comparisons have also characterized how instrument type and collision energy affect spectral similarity for small sets of reference standards.^5,17^ However, the effect of acquisition- and search-parameters on MS/MS searching at the repository scale has not yet been investigated.

MS/MS searching at the repository level recently became available with the introduction of MASST (MAss Spectrometry Search Tool), which enables an MS/MS spectrum to be queried against the Global Natural Products Social molecular networking (GNPS)/MassIVE repository.^11,13^ Unlike MS/MS comparison within a single study, repository-scale searches compare spectra acquired across diverse instruments, laboratories, fragmentation conditions and sample types. Parameter settings that perform well in individual data sets may not generalize to heterogeneous conditions encountered in large public repositories.^18^ MASST is analogous to Basic Local Alignment Search Tool (BLAST), the workflow used for genomic sequence similarity matching.^19^ However, unlike genomic-based searches, which typically report an FDR alongside each match, standard MS/MS search tools provide no equivalent measure of confidence, leaving filtering decisions entirely to user judgment.

Therefore, this study examines MS/MS spectral matching of 40 compounds with diverse chemical structures using a variety of acquisition and analysis parameters. The goal of this work is to provide users with a framework toward best practices for repository-scale MS/MS searching (Figure 1c). Here, we test how acquisition parameters (collision energy, sample concentration and ionization mode) affect MASST search results and estimate false discovery rates across combinations of user-defined analysis thresholds (cosine values and minimum number of matching fragment ions). Together, these results provide a framework for estimating confidence in repository-scale MS/MS matches and underscore the importance of careful parameter selection as queries continue to increase in scale and heterogeneity.

## Methods

This work examines the effect of user-defined parameters on MS/MS spectra and repository-scale matching for a curated library of metabolites, spanning ten chemical classes. Compounds were chosen based on their availability and known prevalence in biological systems. Moreover, this spectral library was designed to include diverse and commonly encountered architectures in metabolomics data sets, namely lipid, steroidal, peptidyl, glycosidic and phenolic structures, across a range of *m*/*z* values (Table S1). MS/MS fragmentation was performed with a Thermo Exploris 120 Orbitrap mass spectrometer for each chemical standard using seven different normalized collision energies (NCEs) from 10 to 70% at 10% intervals. Spectra from a subset of these metabolites were also examined at more granular NCEs (5% intervals), at varying concentrations (1 nM to 10 μM) and in negative ionization mode. All resulting spectra were searched against public metabolomics data using MASST, which queries MS/MS spectra against data indexed by GNPS2 from several public repositories, including MassIVE, Metabolomics Workbench, and MetaboLights. Search results were downloaded and analysis parameters (i.e., minimum cosine values and minimum matching fragment ions) were changed during subsequent filtering steps to explore their effects on repository-scale matching. Search results are reported in two ways: (1) as the total number of spectral matches, which indicates how many unique files the MS/MS were detected in, and (2) the percent of total matches per compound, where each value represents how effective the condition was at maximizing hit rate.

### Sample Preparation for Chemical Standards

Samples for chemical standards were prepared by first dissolving 1 mg of dried material in 1 mL of 1:1 mixture of isopropyl alcohol/ethyl acetate. Then, a 10 μL aliquot was diluted in 50% MeOH/H_2_O to afford a 10 ppm solution. For nonpolar compounds, 0.5 mL of chloroform was added to the initial stock solution to aid in solubility, so 15 μL was transferred to create the 10 ppm solution. For concentration specific runs, 10 μmol of each pure compound was dissolved in 1 mL of 50% isopropyl alcohol/ethyl acetate for the initial stock solution. Serial dilutions with 50% MeOH/H_2_O solution were then performed to afford final concentrations of 10 μM, 1 μM, 100 nM, 10 nM and 1 nM for analysis. All suppliers for commercial standards can be found in Table S1. Protocols for synthetic standards are detailed in the Supporting Information. Reference standards for kurstakins were characterized in a previous study and obtained from the Mevers Lab at Virginia Tech.^20^

### LC–MS/MS Data Acquisition

Chromatographic separation was performed on a polar C18 column (Kinetex Polar C18, 100 mm × 2.1 mm, 2.6 μm particle size, 100 Å pore size, Phenomenex) using a Vanquish UHPLC system coupled to an Exploris 120 Orbitrap mass spectrometer (Thermo Fisher Scientific). The mobile phase consisted of solvent A (water + 0.1% formic acid) and solvent B (ACN + 0.1% formic acid) and the column compartment was kept at 40 °C. A 5 μL amount was injected for each sample and eluted at a flow rate of 0.5 mL/min using the following general gradient: 0–1.0 min 5% B, 1.0–7.0 min 75% B, 7.0–8.0 min 100% B, 8.5–9.5 min 100% B, 9.5–9.6 min 5% B, 9.6–11.0 min 5% B. A different gradient was used for ceramide to accommodate its increased hydrophobicity as follows: 0–1.0 min 5% B, 1.0–7.0 min 75% B, 7.0–8.0 min 100% B, 8.5–13.5 min 100% B, 13.5–13.6 min 5% B, 13.6–15.0 min 5% B.

Mass spectrometry (MS) analysis was performed using heated electrospray ionization (HESI) in positive ionization mode and a subset of metabolites were also subjected to negative mode. All USIs for the curated spectral library can be found in Table S1 and a supplementary .mgf file is also provided with peak list info on GitHub. The parameters were set as follows: sheath gas to 50, auxiliary gas at 10, sweep gas at 2, spray voltage to 3.5 kV (positive) or 2.5 kV (negative), ion transfer tube to 350 °C and vaporizer to 150 °C. An MS scan range from *m*/*z* 100 to 1500 was used with an expected peak width of 6 s and resolution of 60,000 with 1 microscan. The automatic gain control (AGC) target was set to 1 × 10^6^ with a maximum injection time of 100 ms. A targeted mass inclusion list was provided, and dependent scans were performed on up to the 4 most intense ions per MS1 if no target was detected. MS/MS were acquired with a resolution set to 15,000 with 1 microscan, a maximum injection time of 100 ms and an AGC target of 1 × 10^5^. The isolation window was set to 2 *m*/*z* and the isolation offset was turned off. Normalized collision energies between 10 and 70% were used for collection and are listed with each MS/MS spectrum in Table S1. The library is provided as a .mgf file on GitHub and can also be found at this GNPS job link: https://gnps2.org/status?task=05be2b0c9bcd4cf8871251c4236c42eb.

### LC–MS/MS Data Analysis

Raw LC–MS/MS files were converted to .mzML format with MSConvert (ProteoWizard, Palo Alto, CA, USA) and uploaded to GNPS2 for subsequent data processing.^11,21,22^ Using the MS/MS-Chooser workflow, spectra of all desired products were extracted from the .mzML files using molecular SMILES inputs.^23^ This process also designated each MS/MS with a unique spectrum identifier (USI) output (Table S1), which was used in subsequent searching.^24^

MS/MS spectra of the compounds of interest were searched against public metabolomics data files indexed by GNPS2 using MASST.^13^ Specifically, USIs of the verified MS/MS spectra were input into fast MASST (FASST), searching the fragmentation spectra against all public data in GNPS2. All searches were performed using *metabolomicspanrepo_index_latest* in July 2026, and their unfiltered results were downloaded as individual .tsv files. These files were then combined and filtered to parameters outlined in this work, with cosine scores from 0.7 to 1.0 and minimum number of matching peaks from 2 to 6. MASST/FASST results provide search results with a ±1 Da precursor ion tolerance. Therefore, prior to downstream data analysis, candidate matches were manually filtered to a precursor ion tolerance of 0.02 Da, which is the recommended threshold for high-resolution searches to capture matches in qTOF and Orbitrap data.^25^ Results from FASST searches were compiled, removing the scan numbers and duplicate files, to provide lists of unique file matches for each compound. This data was combined into a table that displays total GNPS2 matches. For each compound, total matches is the number of unique repository files that match to the MS/MS spectrum at any collision energy, after applying the cosine and minimum matched peak (MMP) thresholds. The percentage of matches at a given NCE is the fraction of these unique matches that were recovered by the spectrum acquired at that energy. Results were visualized using R and all code used to produce figures can be found on GitHub (https://github.com/keysaj11/MSMS-Matching).

A decoy MS/MS library was generated from the spectra collected in this study in positive ionization mode using the passatutto tool in SIRIUS (v5.8.7), which produces decoy MS/MS spectra from fragmentation trees.^14,26^ Real and decoy spectra were then searched against public data using identical parameters in MASST. For each combination of cosine similarity threshold and minimum matching peaks (MMP), false discovery rate (FDR) was calculated as

FDR=(numberofdecoyspectrawith≥1passingMASSThit)/(numberofrealspectrawith≥1passingMASSThit)×100%

## Results and Discussion

MS/MS spectra were obtained for a library of 40 unique metabolites, which includes sugars, fatty acids, amino acids, small peptides, acylcarnitines, sphingolipids, bile acids, steroid hormones, phenolic compounds and glycosylates, and searched against public metabolomics data using MASST. Each metabolite was subjected to normalized collision energies from 10 to 70%, and a subset was analyzed at different concentrations to assess spectral similarities across these conditions. While concentration is essential for good signal-to-noise ratios, MS/MS spectra are similar once threshold concentration has been reached, as automatic gain control normalizes ion population entering the mass analyzer once sufficient signal is achieved.^27^ In this study, we observed cosine scores greater than 0.98 when comparing spectra collected at concentrations of 100 nM and higher. However, collision energy had a much larger impact on spectral character. MS/MS spectra taken at the most extreme NCEs of 10% versus 70% had cosine values below 0.7 for all compounds (Figure 2a), which is the default cutoff in GNPS platform to define a spectral match. Spectra acquired between 10 and 20% NCEs had low spectral similarity to other spectra for the same molecule (Table S2 and Figure S1). Generally, spectra collected at NCEs of 40% or greater for the same compound were above the cosine threshold of 0.7 when compared to each other, meaning that spectra taken at or above this collision energy are similar to one another and therefore, preferable for repository-scale MS/MS matching (Figure 2a).^28,29^

**Figure 2:**
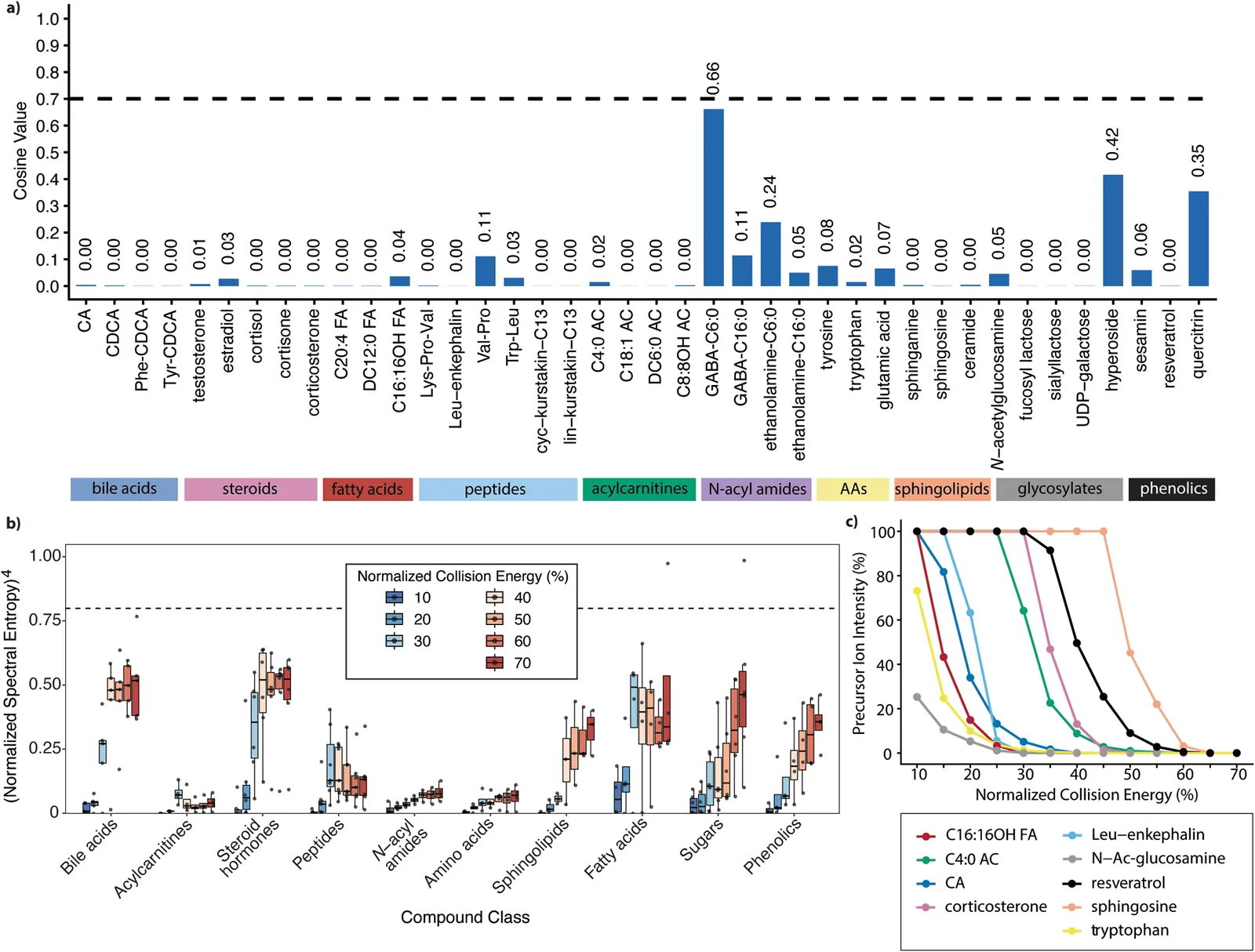
Evaluation of MS/MS spectral library across varying normalized collision energies. (a) Cosine scores between spectra taken at 10 and 70% NCEs for each reference standard. A cosine value of 0.7 is indicated by a dotted line as reference to the default value in GNPS2. (b) (Normalized entropy)^4^ for tested compound classes across different collision energies. The dotted line signifies the recommended maximum cutoff value of 0.8 for high-quality spectra. (c) Connected scatter plot showing the relative intensities of precursor ions versus NCE for a subset of reference standards.

To further assess the impact of collision energies on MS/MS properties, spectral entropies were also calculated for input MS/MS (Figures 2b and S2). Entropy values are useful metrics to approximate the character of MS/MS spectra, where low entropy reflects a spectrum dominated by one or a few intense fragment ions and high entropy indicates a more evenly distributed set of fragment ion intensities. Higher entropies generally reflect more structurally informative fragmentation, though values above recommended upper limits can indicate low-quality, noisy spectra.^15^ Each chemical class showed unique behavior in terms of spectral entropy across collision energies. Compounds with few dominant fragment ions (i.e., amino acids, *N*-acyl amides, acylcarnitines) produced relatively low entropies across all collision energies tested. Metabolites with complex but ultimately convergent fragmentation pathways (i.e., steroidal compounds, fatty acids and peptides) exhibited higher entropy values that peak between 30 and 50% NCEs. Additionally, sphingolipids, sugar-containing metabolites and phenolic compounds possessed a unique pattern, where entropy continually increases as NCE is raised, signifying that these compound classes have divergent fragmentation pathways that are unlocked with increasing collision energies. To further characterize fragmentation behavior, the relative intensity of the precursor ion was then inspected against collision energy for a subset of metabolites (Figure 2c), revealing that although the onset of fragmentation varies substantially by compound class, each compound typically transitions from intact to fully fragmented within a 20% window. Taken together, normalized collision energies between 30 and 50% appear to occupy a “Goldilocks” range for MS/MS spectra, where there is enough fragmentation to yield structural information, without destroying the core molecular architecture. Given these results, we speculated that this collision energy range may also be optimal for downstream MS/MS matching.

To determine optimal values for analysis filtering criteria, we calculated false discovery rates using a fragmentation-based decoy library created in SIRIUS (Figure 3a).^14,16,26,32^ From this analysis, five and six minimum matching peaks (MMP) resulted in <5% FDR at all cosine values tested (0.7–1) while four MMP required cosine values greater than 0.75 to attain this same level of accuracy. When narrowing tolerance to 1% FDR, six MMP remained reliable across the entire cosine range while four MMP required cosine values of ∼0.9 or greater. Spectra with only three major fragment ions should be searched with caution, but can be queried with <5% FDR at cosine values above 0.85 and with 1% FDR when cosine scores are ≥0.9. Two fragment ions cannot be reliably used, as this setting did not produce <5% FDR until cosine thresholds were higher than 0.9. These results highlight the importance of the minimum number of matching fragment ions as a filtering parameter in repository-scale matching and demonstrates how FDR can be controlled in MASST searches through appropriate selection of user-defined thresholds. To maintain false discovery rates of <5%, results were filtered to 4 or 6 MMP and 0.8 or 0.7 minimum cosine values, respectively, in this study.

**Figure 3:**
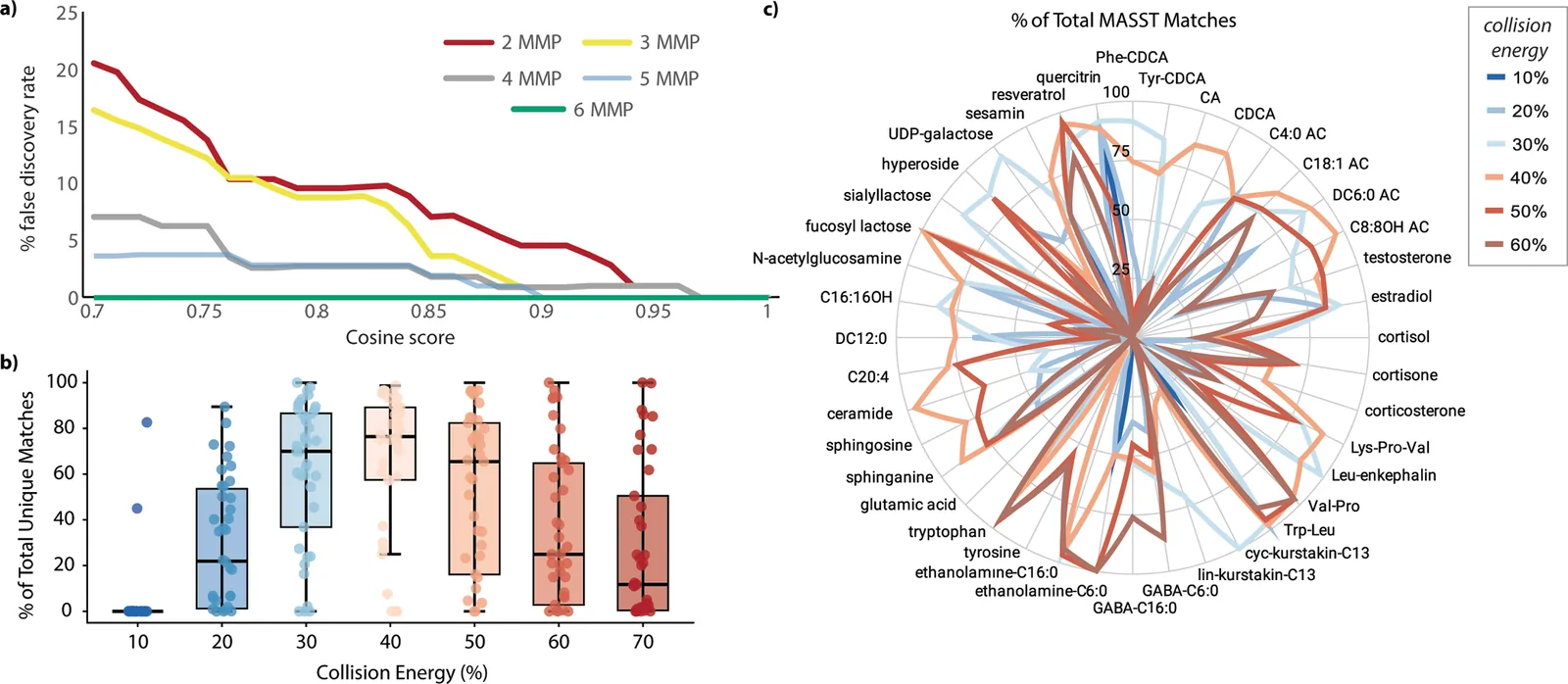
Comparison of MASST search results for a reference library of 40 compounds. (a) False discovery rates for different minimum matching peaks filtered across tested cosine values. (b) Boxplots illustrating the proportion of total matches obtained at varying normalized collision energies, where each jitter represents a compound. Four MMP and 0.8 cosine score cutoffs were used in this comparison, and the same trend was also observed with other threshold values. (c) Radar plot showing the % of MASST matches achieved at varying NCEs per individual metabolite using four MMP and 0.8 cosine score cutoff.

Considering the differences observed for MS/MS spectra of the same molecule when taken under varying conditions, we next explored how acquisition factors impact MS/MS searching at the repository scale using MASST. Previous work already examined the effects of collecting input MS/MS on different instruments (QToF versus Orbitrap) for spectral matching, finding that MASST search results are similar between detectors.^30^ Therefore, this study focused on testing the effects of collision energy on repository-scale spectral matching, as this was shown above to have a large impact on spectral character. In support of our previous hypothesis, normalized collision energies around 40% were determined to be optimal for MASST searching, as the largest proportion of total matches to public data files are obtained at this setting (Figure 3b). In terms of absolute collision energies, midrange values between 20 and 60 eV were preferable for our searches, providing a higher proportion of total matches than extreme values (Figure S3). As was observed for cosine similarity and spectral entropy, this midrange value provides enough energy to fragment most compounds sufficiently without eliminating larger fragments. The majority of tested compounds achieved 75% or more of their total matches using only spectra acquired at 40% NCE (Figure 3c, Tables 1 and S3). Amino acids and peptides were the only outliers observed that required other collision energies to maximize search results, though this is structure dependent (Figure S4). For example, when results are filtered to four MMP, tryptophan and Trp–Leu provide >50% of matches at all NCEs ≥ 20%, whereas glutamic acid and Val–Pro require 70% and ≥40% NCEs, respectively, to achieve match results. Relative intensity of the precursor ion was also compared to the proportion of total matches to see if a correlation could be observed, but there was no obvious relationship detected, which suggests that collision energy matters more than precursor ion intensity for MASST searches (Figure S5). The effect of collecting data in positive or negative electrospray ionization mode was also tested on a subset of metabolites. For compounds that can be detected well in either ionization modes (e.g., cholic acid, *N*-acetylglucosamine), more MASST matches were obtained in positive mode, which likely reflects a bias toward positive mode data in the repository (Figure S6).^31^

**Table 1:** Selected Data from Tables S1 and S3 Showing *m*/*z* Values and MASST Search Results for the Metabolite Library Across Varying Normalized Collision Energies and Filter Parameter Values^a^

				cosine = 0.70, MMP = 6				cosine = 0.80, MMP = 4			
					% of matches at each NCE				% of matches at each NCE		
class	metabolite	adduct	*m*/*z*	total matches	20%	40%	60%	total matches	20%	40%	60%
bile acids	Phe-CDCA	M + H^+^	540.3684	3645	77.4	93.2	1.8	2917	35.7	74.6	0.4
	Tyr-CDCA	M + H^+^	556.3633	1248	65.5	86.4	0.5	795	19.0	70.2	0.0
	CA	M – 2H_2_O + H^+^	373.2737	45618	0.7	92.8	27.7	29132	0.1	85.7	21.0
	CDCA	M – 2H_2_O + H^+^	357.2788	58712	31.4	90.5	6.7	38164	6.8	87.2	2.8
acylcarnitines	C4:0 AC	M+	232.1543	0	0.0	0.0	0.0	25994	72.9	72.5	25.2
	C18:1 AC	M+	426.3583	17847	29.3	48.3	25.3	36127	18.3	86.1	70.8
	DC6:0 AC	M+	290.1598	14928	91.6	96.6	42.3	15364	62.0	93.2	49.8
	C8:8OH AC	M+	304.2118	4446	17.4	70.4	0.0	13151	22.4	96.6	24.9
steroid hormones	testosterone	M + H^+^	289.2162	148	4.1	64.9	2.0	309	54.7	81.9	61.8
	estradiol	M + H^+^	273.1849	88	14.8	35.2	1.1	17	82.4	82.4	52.9
	cortisol	M + H^+^	363.2166	7486	56.0	80.1	10.8	5644	35.0	37.2	28.2
	cortisone	M + H^+^	361.2010	1297	18.0	81.5	61.9	1091	5.9	60.6	64.8
	corticosterone	M + H^+^	347.2217	2513	33.8	94.1	2.1	2130	4.7	58.7	17.4
peptides	Lys-Pro-Val	M + H^+^	343.2340	119	96.6	65.5	0.0	121	36.4	90.1	38.8
	Leu-enkephalin	M + H^+^	556.2771	320	26.3	88.8	17.8	302	21.5	88.1	14.9
	Val–Pro	M + H^+^	215.1390	55	0.0	5.5	0.0	20948	0.6	95.7	96.6
	Trp–Leu	M + H^+^	318.1820	12435	89.6	90.4	95.4	14181	95.7	97.9	89.3
	cyc-kurstakin-C13	M + H^+^	906.5043	13	0.0	46.2	0.0	4	0.0	25.0	0.0
	lin-kurstakin-C13	M + H^+^	924.5149	12	0.0	25.0	0.0	10	0.0	30.0	0.0
*N*-acyl amides	GABA-C6:0	M + H^+^	202.1438	0	0.0	0.0	0.0	1107	40.2	57.5	85.7
	GABA-C16:0	M + H^+^	342.3003	341	0.0	88.3	13.2	1247	35.7	51.0	76.1
	ethanolamine-C6:0	M + H^+^	160.1332	0	0.0	0.0	0.0	8	50.0	50.0	100
	ethanolamine-C16:0	M + H^+^	300.2897	14849	0.0	0.0	68.6	35997	0.0	93.5	93.7
amino acids	tyrosine	M + H^+^	182.0812	171	0.0	0.0	87.7	78900	2.4	5.5	60.4
	tryptophan	M + H^+^	205.0972	99626	0.4	35.7	67.4	146678	54.9	75.2	93.6
	glutamic acid	M + H^+^	148.0604	0	0.0	0.0	0.0	1701	0.0	0.0	0.0
sphingolipids	sphinganine	M + H^+^	302.3054	9865	1.1	20.4	34.1	16596	49.5	89.2	67.2
	sphingosine	M – H_2_O + H^+^	282.2791	12315	0.0	64.9	10.1	17118	0.0	79.0	37.9
	ceramide	M + H^+^	538.5194	15525	0.0	88.1	18.7	29965	40.5	96.6	15.0
fatty acids	C20:4 FA	M + H^+^	305.2475	26757	6.5	86.9	4.7	19950	1.9	79.4	6.0
	DC12:0 FA	M + H^+^	231.1591	2867	65.1	89.3	18.4	2831	67.6	75.0	6.7
	C16:16OH FA	M – H_2_O + H^+^	255.2319	33418	8.0	83.4	2.8	26049	1.0	77.3	21.0
sugars	*N*-acetylglucosamine	M + H^+^	222.0972	3974	0.0	67.2	26.1	9690	72.2	76.0	8.2
	fucosyl lactose	M + Na^+^	511.1633	549	0.0	89.1	2.0	608	0.0	98.7	0.0
	sialyllactose	M + H^+^	634.2189	2680	61.3	47.2	0.0	1779	6.7	7.5	0.0
	hyperoside	M + H^+^	465.1028	6331	0.0	21.0	75.6	9787	63.6	64.6	32.4
	UDP-galactose	M + Na^+^	589.0442	636	49.2	48.7	0.2	560	50.2	27.1	0.2
phenolics	sesamin	M – H_2_O + H^+^	337.1081	452	77.0	87.4	84.1	411	56.9	76.4	58.6
	resveratrol	M + H^+^	229.0859	2057	0.0	78.1	21.8	2765	0.0	95.2	79.9
	quercitrin	M + H^+^	449.1078	1483	0.0	35.8	69.9	3911	89.4	89.4	20.2

a Total matches are unique repository files matched at any collision energy. % of matches represents the portion of total matches recovered by the spectrum acquired at that NCE.

The greatest number of matches were obtained for amino acids, bile acids and acylcarnitines (Table S3). Tryptophan was the most frequently detected metabolite in the library, returning nearly 150,000 unique matches after filtering with a minimum of 4 matching fragment ions and cosine value of 0.8. Chenodeoxycholic acid and tyrosine were also frequently detected in public data, with over 50,000 hits each at their respective thresholds of 6 minimum matching peaks with 0.7 cosine score and 4 minimum matching peaks with 0.8 cosine value. This analysis found that compounds with higher spectral entropies (i.e., bile acids, steroids, fatty acids) perform reliably with six minimum matching peaks, but those with spectral entropies at or below two (i.e., amino acids, dipeptides, acylcarnitines, *N*-acyl amides) (Figure S2), require fewer minimum matching fragment ions to obtain matches to public data. The total numbers of spectral matches across chemical subclasses are relatively consistent in the cosine value range between 0.7 and 0.9, especially when at least six minimum fragment ions were matched, then drop off as cosine values approach one (Figures S7 and S8). The absence of a sharp inflection point makes it impossible to identify the cosine threshold at which false positives appear from match counts alone, so FDR calculations were essential for determining acceptable match parameters.

There are additional factors that must also be considered in repository-scale MS/MS matching. First, input MS/MS data should be from high resolution instruments given the narrow mass tolerances (≤0.02 Da) used in these searches. Additionally, the chemical structure of a metabolite and its environment can bias observation in LC–MS/MS data sets and lead to false negatives for several reasons. Some compounds simply do not ionize well in positive or negative modes and require millimolar or higher concentrations to detect by MS/MS, which will not be achieved in biological samples.^33^ Matrix effects can also bias detection, where some molecules may not appear in public data due to ion suppression from coeluting metabolites that overpower the signal.^34,35^ Moreover, metabolites that are strongly polar or nonpolar can also be missed in standard untargeted acquisition methods because they elute too quickly or late, and can be diverted to waste.^36^ During the electrospray process, adducts other than the [M + H]^+^ ion can also form through metal coordination and in-source fragmentation.^37,38^ In some cases, the alternative adducts may provide better options for MS/MS searching than the expected precursor ion. For example, sugars like *N*-acetylglucosamine undergo in-source losses of water, such that the intact precursor ion is rarely detected while its [M – H_2_O + H]^+^ counterpart is far more prevalent (Figure 4). Failing to search for these adducts can therefore result in false negatives. Finally, even a successful MS/MS match does not guarantee unambiguous identification. Regio- and stereoisomers typically produce indistinguishable MS/MS spectra, so matches cannot serve as confirmation of an exact chemical structure without rigorous downstream validation. Additionally, in-source fragmentation can further complicate compound identification, as fragmentation byproducts can be misannotated as genuine metabolites when they share the same mass as biologically relevant compounds.^39^ With all considerations in mind, users must ultimately assess the confidence level of matching possible from their search based on purity of reference standard, chemical structure of the metabolite and level of confidence based on Metabolomics Standards Initiatives guidelines.^40^

**Figure 4:**
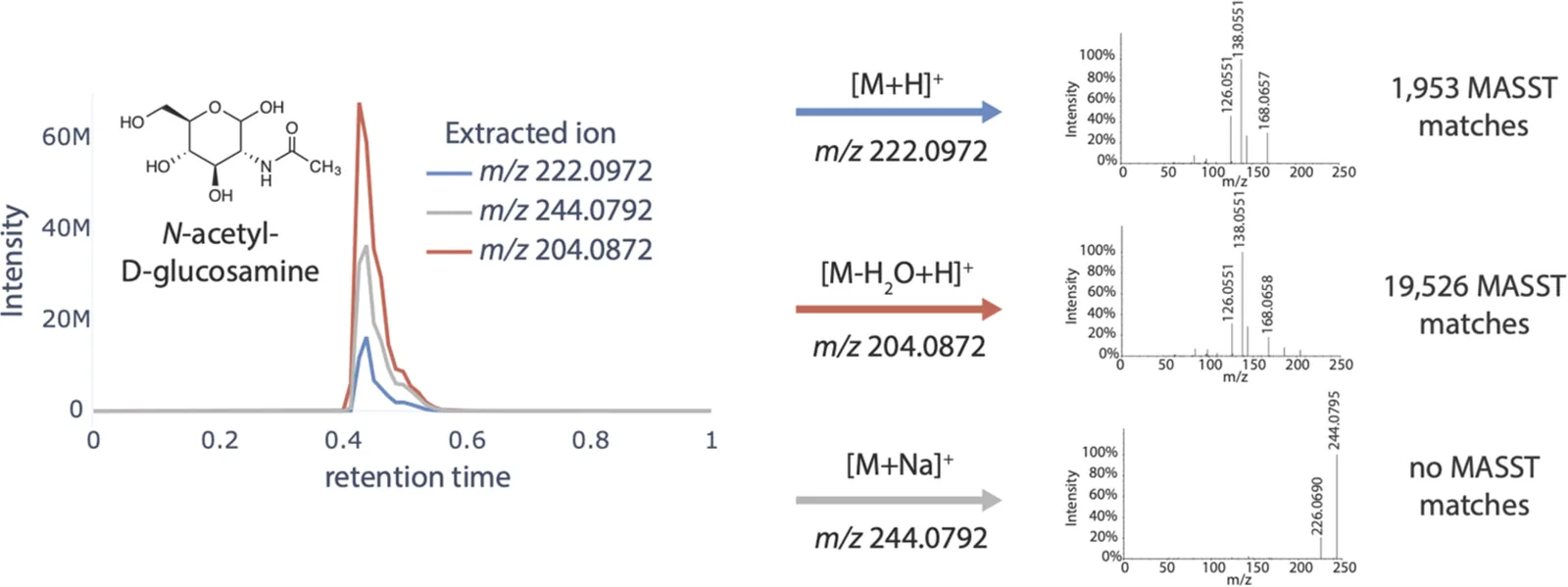
Overlaid XICs for different adducts of *N*-acetylglucosamine along with their resulting MS/MS spectra and respective MASST results in GNPS2.

## Conclusion

This work establishes best practices for repository-scale MS/MS matching, addressing a current gap in knowledge that is of increasing importance to the community. As with genomic sequence comparison, repository-scale spectral matching requires statistical rigor to be trusted as a discovery tool. Taken together, our findings show that midrange collision energies (30–50% NCE, 20–60 eV) produce the most informative MS/MS for spectral searching, and that this same acquisition window enables low false discovery rates (FDR) in MASST searches when paired with appropriate cutoffs for cosine score and minimum number of matching fragment ions. During downstream data analysis, a minimum of six matching fragment ions should be used where possible for consistently low FDR across cosine values of 0.7 and above. Where lower minimum peak counts are unavoidable, such as with acylcarnitines and amino acids that are dominated by few diagnostic fragments, cosine thresholds should be raised accordingly and results interpreted with greater caution.

Our findings also underscore that FDR alone cannot resolve every source of ambiguity in repository-scale matching. Structural isomers remain mostly indistinguishable by MS/MS alone regardless of match confidence. Additionally, other factors like ion suppression, adduct formation and in-source fragmentation can decrease detection of expected metabolites, and database composition itself introduces bias. Finally, although our test set includes recently annotated classes such as kurstakins, conjugated bile acids and *N*-acyl amides, all compounds examined here have well-characterized reference spectra. Extending these guidelines to the unannotated metabolome will require further investigation. As repository-scale searching continues to expand, so must our efforts to minimize false discovery, so that confidence in these results keeps pace with their scale.

## Supplementary Material





## Notes

Table S1: USIs and information for all reference standards; Table S2: cosine score comparisons of spectra collected at varying collision energies; Table S3: MASST results for reference standard library; Table S4: absolute collision energies for collected reference spectra (XLSX)
Supporting Information Additional details about synthetic protocols and Figures S1–S8 (PDF)
