# Supplementary material for: Evaluating the Effects of Acquisition and Search Parameters on Repository-Scale MS/MS Matching
Source: J Am Soc Mass Spectrom. 2026 Oct 23;37(11):2922–30. doi: 10.1021/jasms.6c00332 (PMC13741904; doi:10.1021/jasms.6c00332)
Supplement: Supplementary file 1 [file js6c00332_si_002.pdf]

Supporting Information

# Evaluating the Effects of Acquisition and Search Parameters on Repository-Scale MS/MS Matching

*AUTHOR NAMES*

Allison J. Keys[1], Emily C. Gentry[1]*

AUTHOR ADDRESS

[1] Virginia Tech, Department of Chemistry, Blacksburg, VA, USA, 24061
* correspondence to egentry@vt.edu

**Table of Contents**

**S1.** Synthesis of Select Reference Standards……S3
**S1.1.** Materials……S3
**S1.2.** Acylcarnitines……S3
**S1.3.** Dipeptides……S3
**S1.4.** Bile Acids……S3
**S1.5.** *N*-acyl amides……S3
**Figure S1**……S4
**Figure S2**……S5
**Figure S3**……S6
**Figure S4**……S7
**Figure S5**……S8
**Figure S6**……S9
**Figure S7**……S10
**Figure S8**……S11
**References**……S12

## S1. Synthesis of Select Reference Standards

### *S1.1. Materials*

Organic solutions were concentrated under reduced pressure on a Büchi rotavapor R-205 using a Büchi Heating Bath B-490. All reactions were conducted in a well-ventilated fume hood. Dry dimethylformamide (DMF) and 3,4-dihydropyran were purchased from Thermo Scientific.

### *S1.2. Acylcarnitines*

The respective fatty acids (1 equiv, 50 μmol) and triethylamine (2 equiv, 100 μmol) were dissolved in 250 μL of DMF and allowed to stir at 0 °C for 5 mins. Ethylchloroformate (1.1 equiv, 55 μmol) was then added slowly and the resulting mixture was stirred at 0 °C for 15 mins, then L-carnitine (1.2 equiv, 60 μmol) was added and the reaction was stirred for an hour, warming to room temperature. For C8:8OH, the fatty acid alcohol was protected with 3,4-dihydro-2H-pyran (1.5 equiv, 75 μmol) for 15 mins prior to addition of triethylamine. The reaction mixture was dried and stored at -20 °C until LC-MS/MS data acquisition.

### *S1.3. Dipeptides*

A similar process to the acylcarnitine synthesis was followed, where the respective *N*-Boc-protected amino acids (1 equiv, 100 μmol) and triethylamine (2 equiv, 200 μmol) were dissolved in 1 mL of DMF solution at 0 °C and allowed to stir for 5 mins. Ethylchloroformate (1.1 equiv, 110 μmol) was then added slowly and stirred for 15 mins, then the respective second amino acid (1.5 equiv, 150 μmol) was added and stirred for an hour, gradually warming to room temperature. The reaction mixture was dried and stored at -20 °C until LC-MS/MS data acquisition.

### *S1.4. Bile Acids*

Conjugated bile acids were synthesized using a method reported previously.[1]

### *S1.5. N-acyl amides*

*N*-acyl amides were synthesized by a method adapted from Mullowney *et al*.[2]

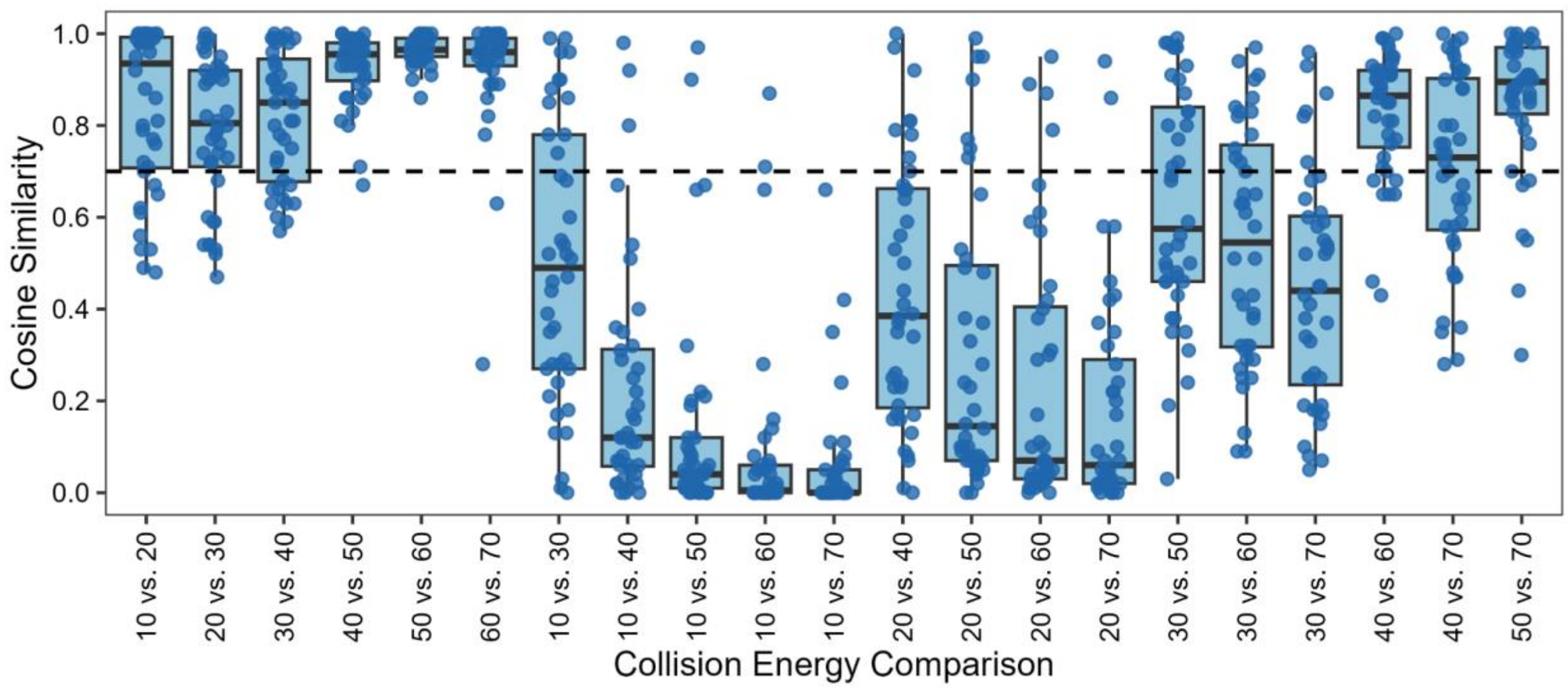


**Figure S1:** Cosine similarity scores between MS/MS spectra taken at varying collision energies.

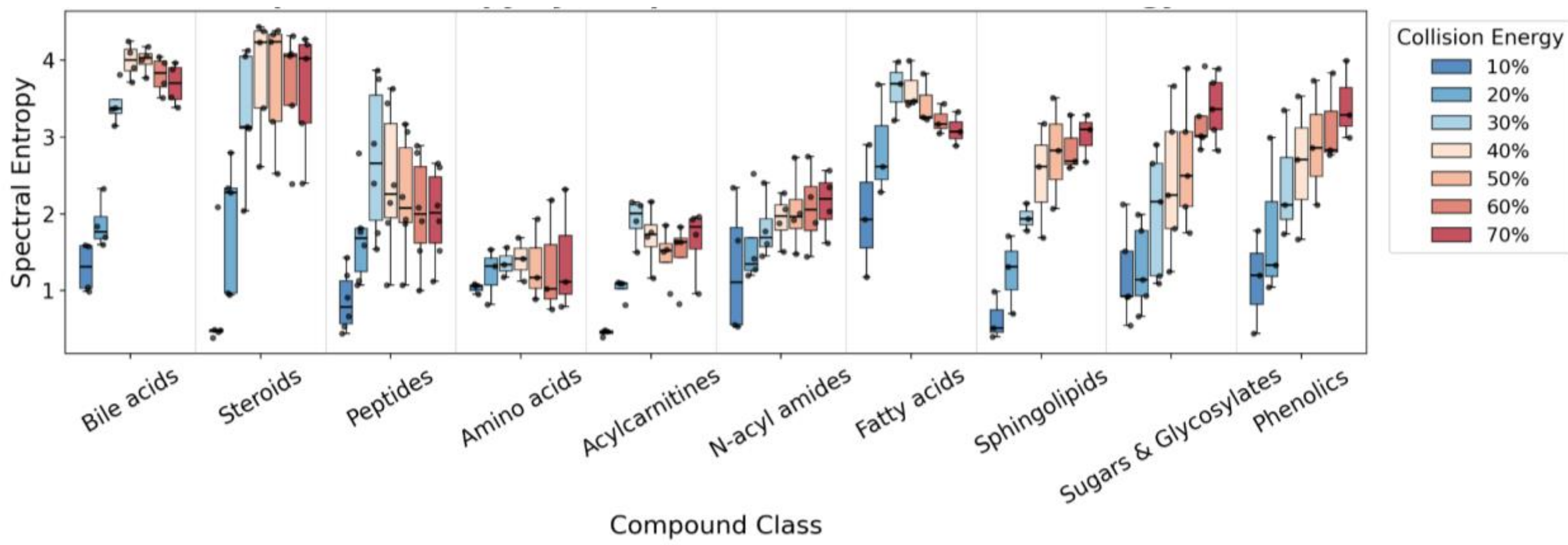


**Figure S2:** Spectral entropies of MS/MS library shown by compound class over varying collision energies.

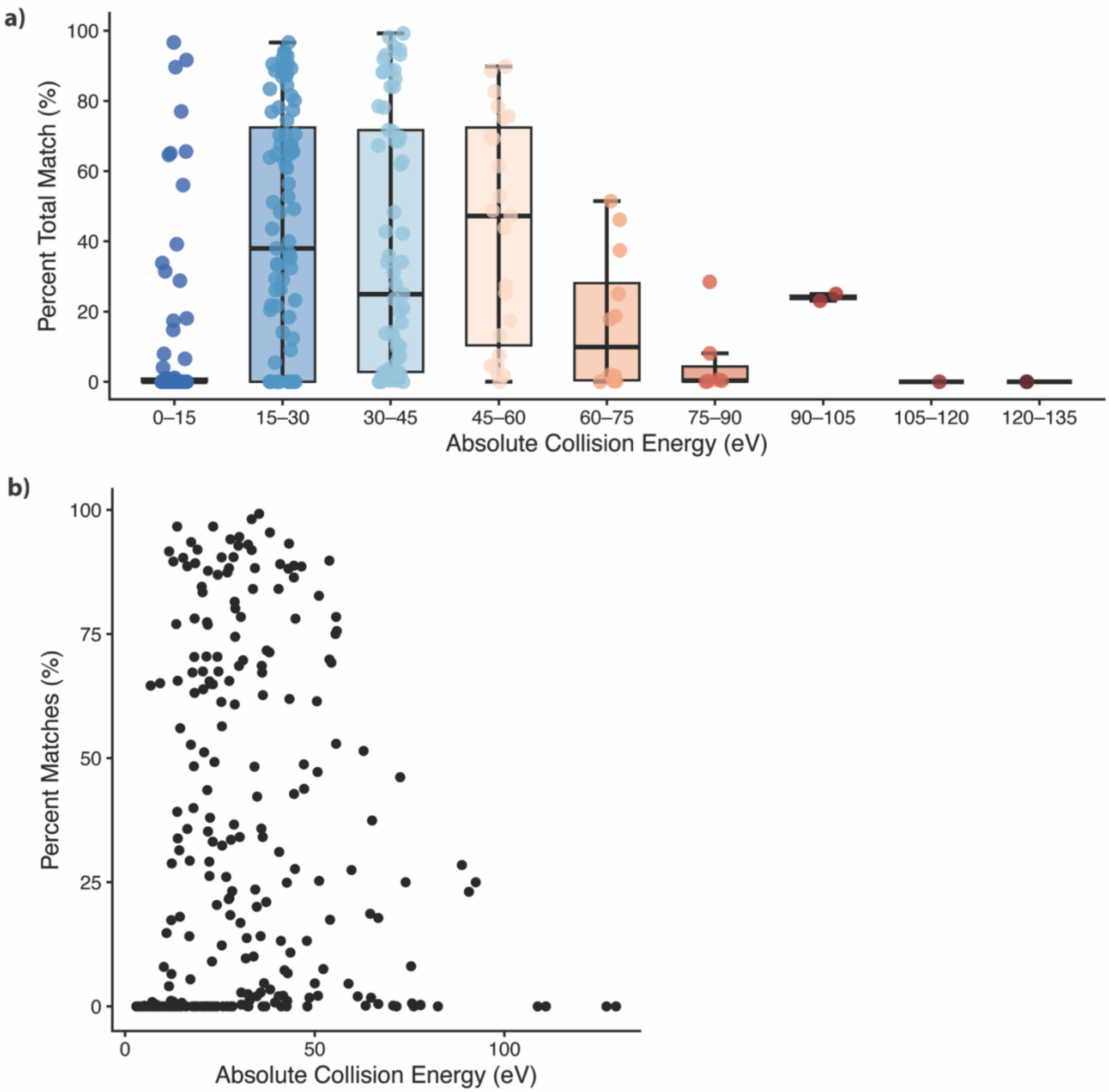


**Figure S3:** Percent of total matches shown by absolute collision energy. **a)** Boxplot illustrating the proportion of total matches obtained at varying absolute collision energies, where each jitter represents a compound. Four MMP and 0.8 cosine score cutoffs were used in this comparison, and the same trend was also observed with other threshold values. **b)** Scatter plot illustrating distribution of matches across collision energies, where the majority of matches are found between 20-60 eV.

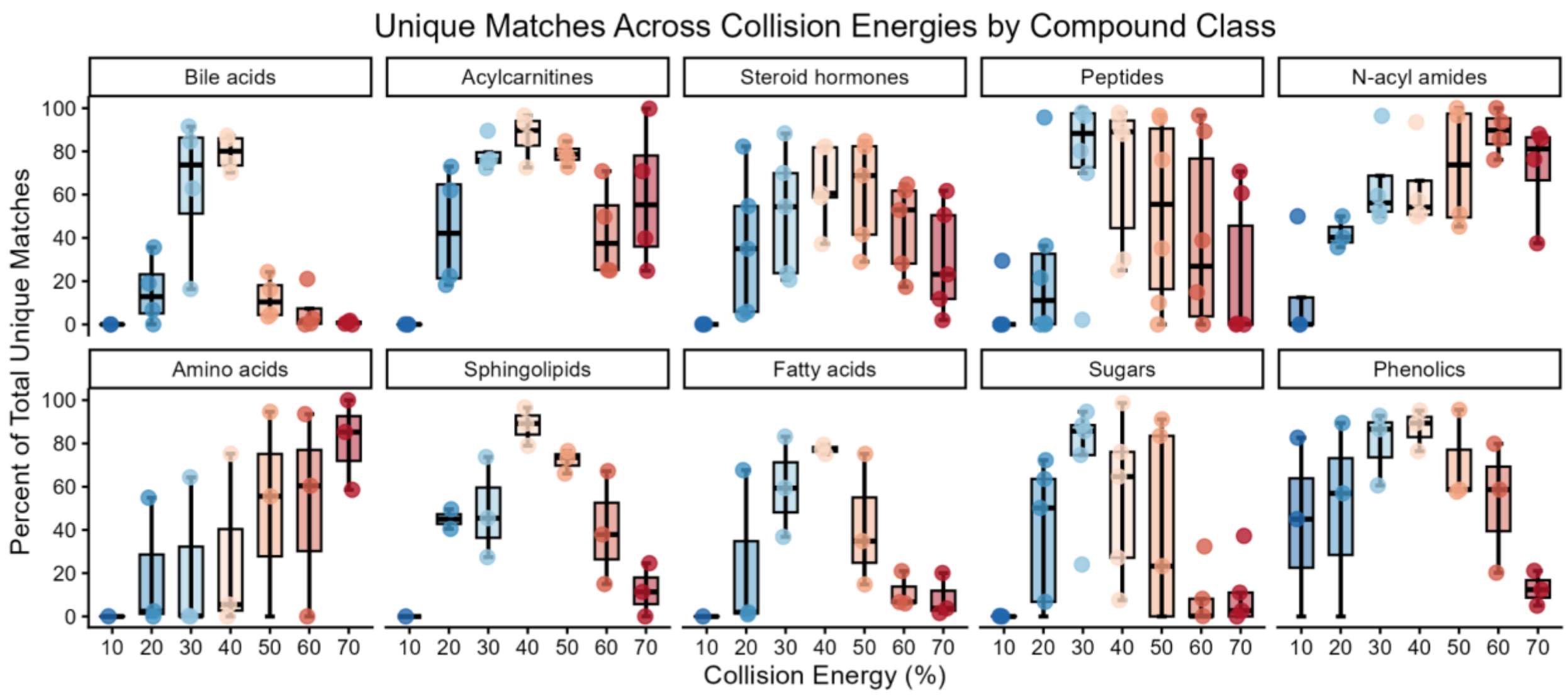


**Figure S4:** Faceted boxplots demonstrating proportion of unique matches at varying collision energies, faceted by compound classes in this study. These matches are filtered to a cosine of 0.8 and four minimum matching peaks.

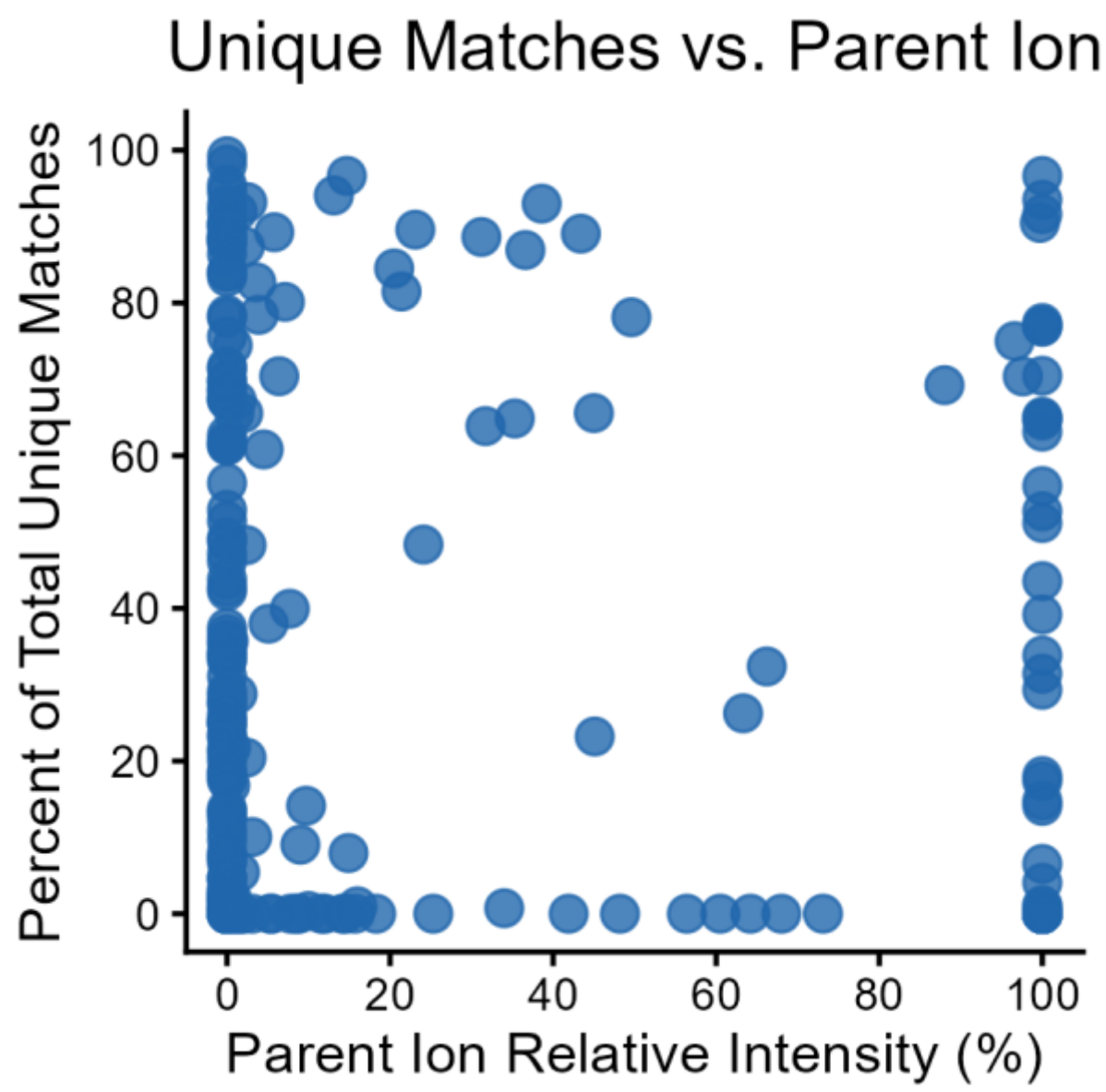


**Figure S5:** Scatter plot of percent unique matches compared to relative intensity of parent ion, where each dot represents a unique compound. These matches are filtered to a cosine of 0.7 and minimum matching peaks of 6. Percent of total unique matches represents the proportion of total match results that a compound has at collision energy collected.

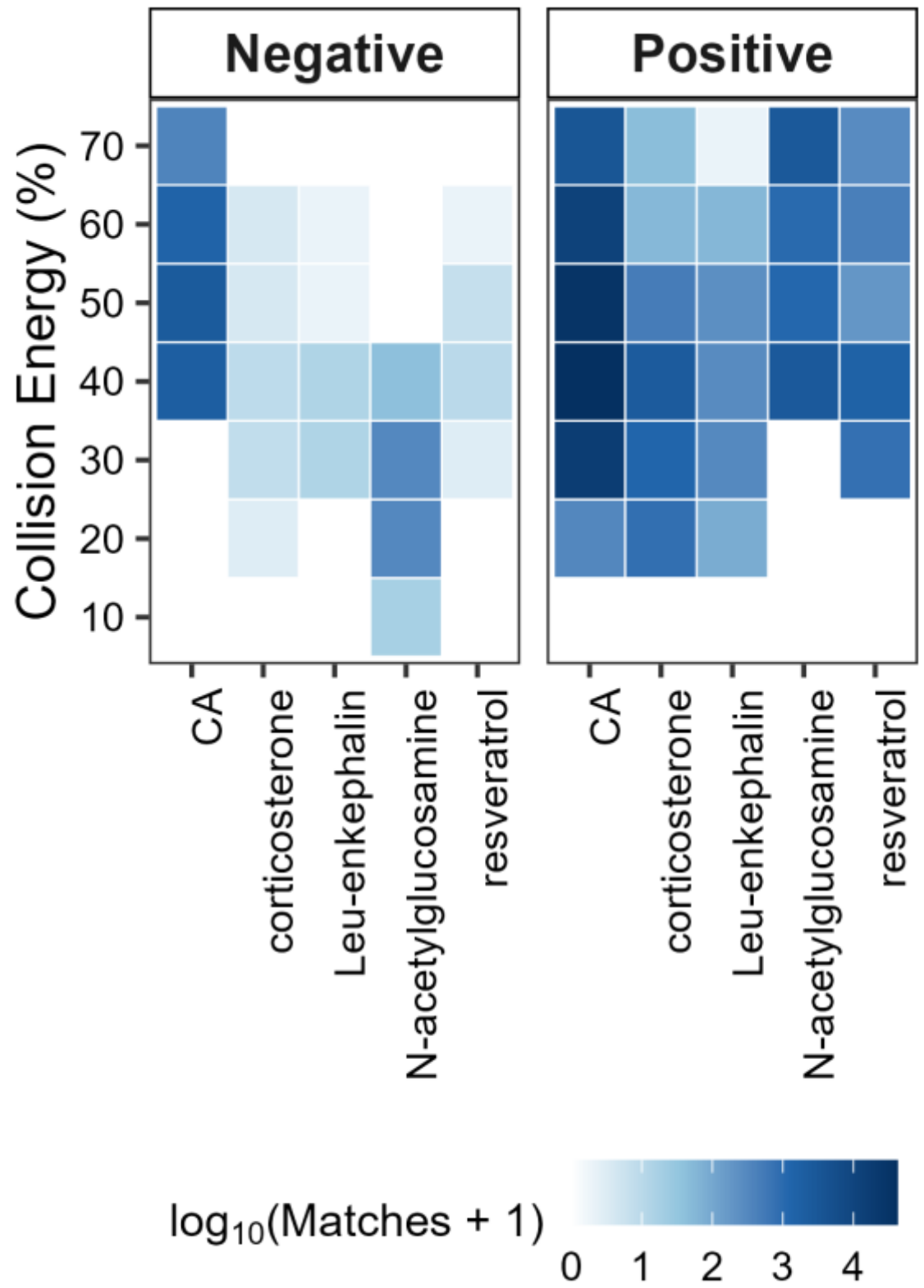


**Figure S6:** Faceted heatmaps comparing the number of matches (logarithmically) for spectra collected in negative and positive ionization mode, across all collision energies collected. These matches are filtered to a cosine of 0.7 and minimum matching peaks of 6. CA is cholic acid.

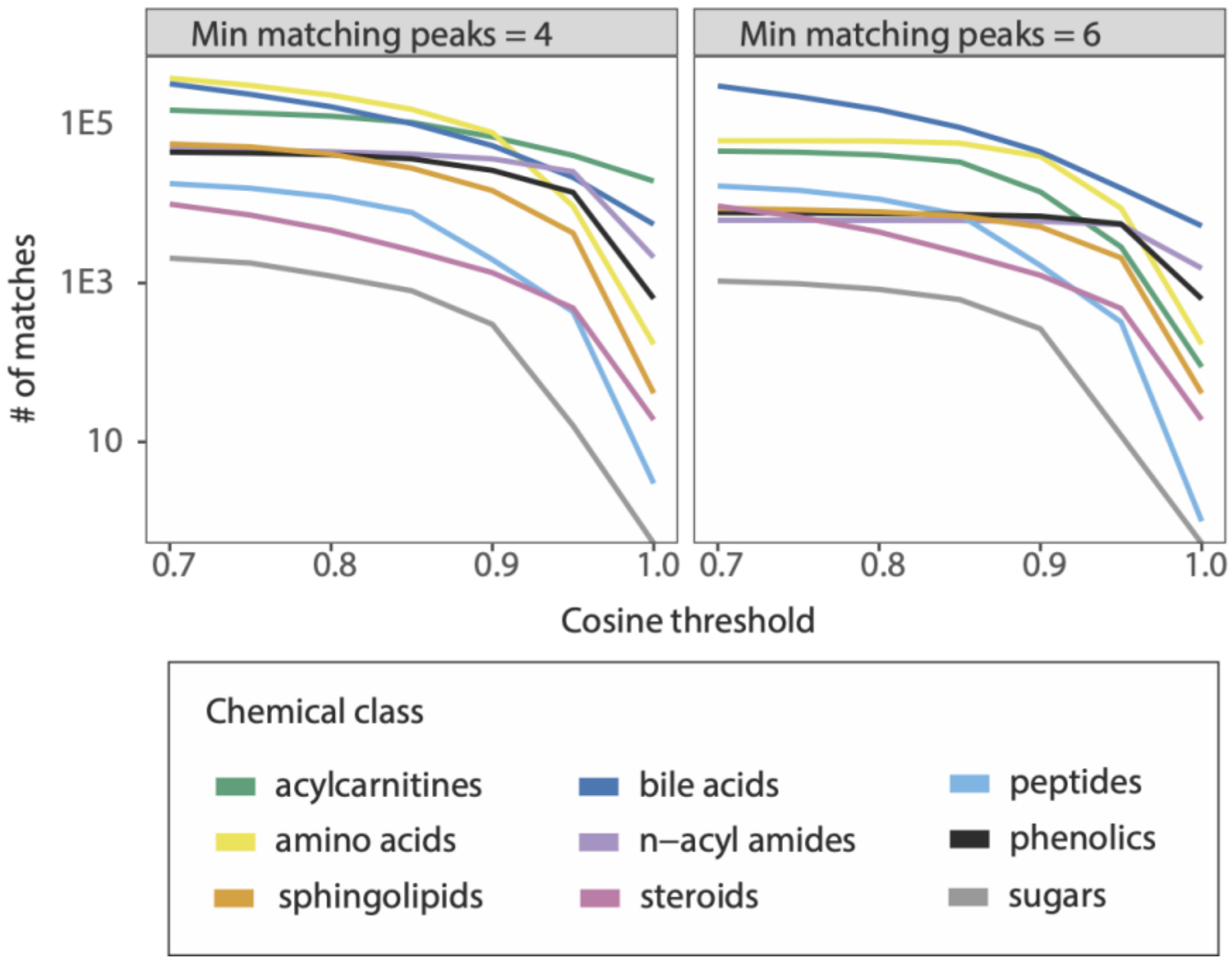


**Figure S7:** Number of MASST matches per compound class across varying cosine thresholds and minimum number of fragment ions.

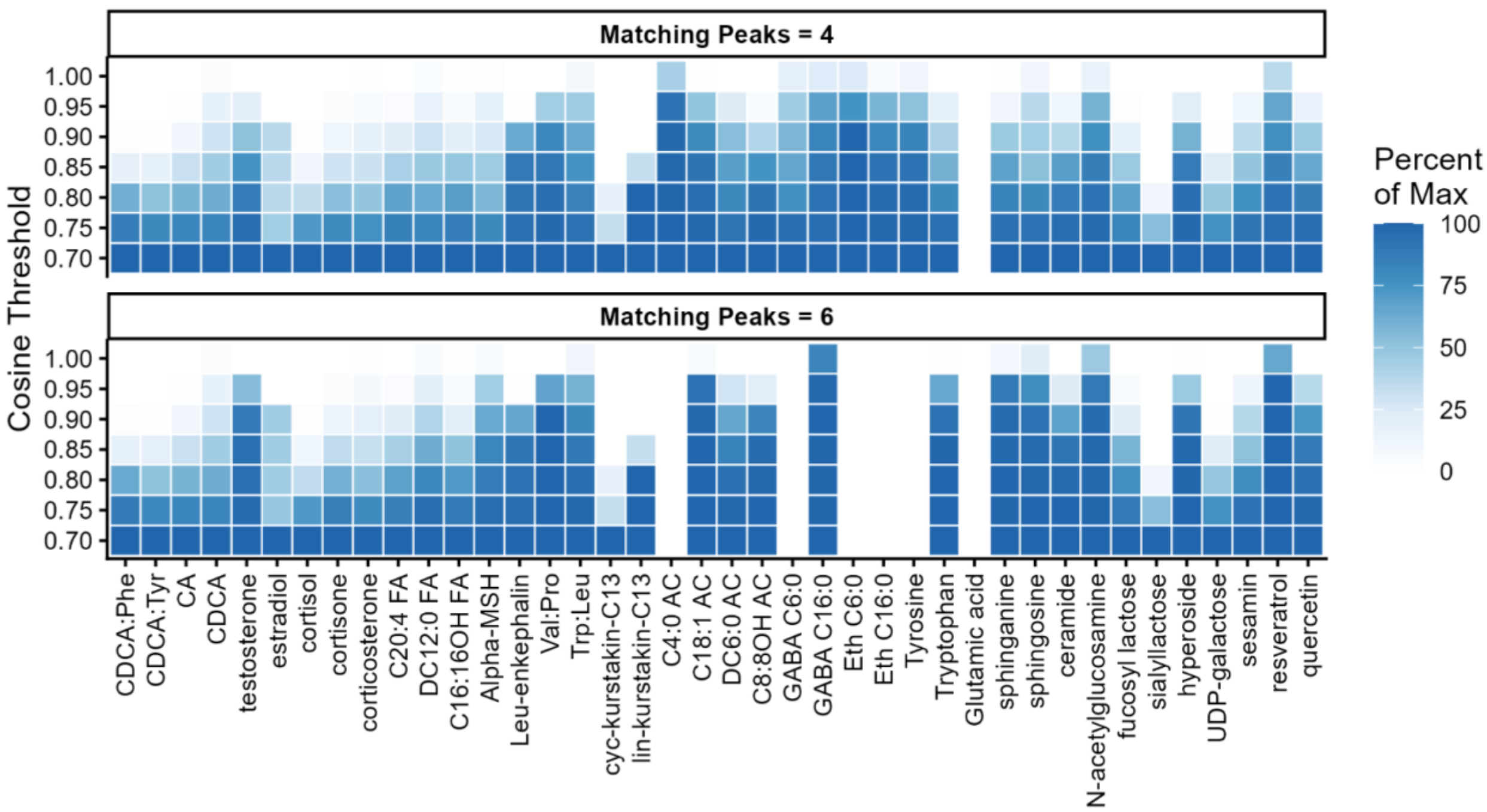


**Figure S8**: Heatmap displaying the percent of total matches at 40% normalized collision energy per compound across various cosine thresholds with four or six minimum matching peaks.

## References

(1) Gentry, E. C.; Collins, S. L.; Panitchpakdi, M.; Belda-Ferre, P.; Stewart, A. K.; Carrillo Terrazas, M.; Lu, H.; Zuffa, S.; Yan, T.; Avila-Pacheco, J.; Plichta, D. R.; Aron, A. T.; Wang, M.; Jarmusch, A. K.; Hao, F.; Syrkin-Nikolau, M.; Vlamakis, H.; Ananthakrishnan, A. N.; Boland, B. S.; Hemperly, A.; Vande Casteele, N.; Gonzalez, F. J.; Clish, C. B.; Xavier, R. J.; Chu, H.; Baker, E. S.; Patterson, A. D.; Knight, R.; Siegel, D.; Dorrestein, P. C. Reverse Metabolomics for the Discovery of Chemical Structures from Humans. *Nature* **2024**, *626* (7998), 419–426. https://doi.org/10.1038/s41586-023-06906-8.

(2) Mullowney, M. W.; Fiebig, A.; Schnizlein, M. K.; McMillin, M.; Rose, A. R.; Koval, J.; Rubin, D.; Dalal, S.; Sogin, M. L.; Chang, E. B.; Sidebottom, A. M.; Crosson, S. Microbially Catalyzed Conjugation of GABA and Tyramine to Bile Acids. *J. Bacteriol.* **2024,** *206* (1), e00426-23. https://doi.org/10.1128/jb.00426-23.
